# Anti-CD47 antibodies induce phagocytosis of live, malignant B cells by macrophages *via* the Fc domain, resulting in cell death by phagoptosis

**DOI:** 10.18632/oncotarget.18492

**Published:** 2017-06-15

**Authors:** Lucy E. Métayer, Anna Vilalta, G.A. Amos Burke, Guy C. Brown

**Affiliations:** ^1^ Department of Biochemistry, University of Cambridge, Cambridge, UK; ^2^ Department of Pediatrics, University of Cambridge, Cambridge, UK

**Keywords:** phagocytosis, cell death, leukemia, antibodies, phagoptosis, Autophagy

## Abstract

When expressed on the surface of cells, CD47 inhibits phagocytosis of these cells by phagocytes. Most human cancers overexpress CD47, and antibodies to CD47 have shown a remarkable ability to clear a range of cancers in animal models. However, the mechanism by which these antibodies cause cancer cell death is unclear. We find that CD47 is expressed on the surface of three B-cell lines from human malignancies: 697 (pre-B-ALL lymphoblasts), Ramos and DG-75 (both mature B-cells, Burkitt’s lymphoma), and anti-CD47 antibodies greatly increase the phagocytosis of all three cell line by macrophages. In the presence of macrophages, the antibodies cause clearance of the lymphoblasts within hours, but in the absence of macrophages, the antibodies have no effect on lymphoblast viability. Macrophages engulf viable lymphoblasts containing mitochondria with a normal membrane potential, but following engulfment the mitochondrial membrane potential is lost indicating a loss of viability. Inhibition of phagocytosis protects lymphoblasts from death indicating that phagocytosis is required for anti-CD47 mediated cell death. Blocking either the antibody Fc domain or Fc receptors inhibits antibody-induced phagocytosis. Antibodies against cell surface markers CD10 or CD19 also induced Fc-domain-dependent phagocytosis, but at a lower level commensurate with expression. Thus, phagoptosis may contribute to the efficacy of a number of therapeutic antibodies used in cancer therapy, as well as potentially endogenous antibodies. We conclude that anti-CD47 antibodies induce phagocytosis by binding CD47 on lymphoblast and Fc receptors on macrophages, resulting in cell death by phagocytosis, i.e. phagoptosis.

## INTRODUCTION

Phagocytosis is the engulfment of large (>0.5 micron) extracellular particles by cells. Cells specialised in phagocytosis are termed ‘phagocytes’, and in mammals include macrophages, monocytes, microglia, neutrophils and dendritic cells [1, 2]. Particles engulfed by phagocytes may include other cells. Cells are phagocytosed by competent phagocytes if they: i) expose on their surface so-called ‘eat-me’ signals, such as phosphatidylserine or calreticulin, ii) lose so-called ‘don’t-eat-me’ signals, such as CD47 or surface sialylation, and/or iii) bind opsonins, such as complement factors or IgG antibodies [1-3]. It has in the past been generally assumed that phagocytes only phagocytose dead or dying cells [2, 3]. However, there are a variety of circumstances in which otherwise viable cells are phagocytosed and thereby killed as a result of the phagocytosis [4]. This type of cell death by phagocytosis, which we have called ‘phagoptosis’, has the defining characteristic that if phagocytosis is inhibited then cell death is prevented [4]. Examples of such phagocytic cell death are: physiological turnover of senescent erythrocytes [5, 6], neutrophil phagocytosis of bacteria [7, 8] and developmental loss of neurons [9, 10].

Recently it has become clear that most human cancer cells overexpress CD47 on their surface apparently to prevent themselves being phagocytosed [11]; their phagocytosis being promoted by the presence of calreticulin on the surface of the cancer cells [12]. Remarkably, if CD47 is blocked by an anti-CD47 antibody then a variety of cancers can be cleared from mice [11-13]. Suggested mechanisms include that the anti-CD47 antibodies: i) induce phosphatidylserine exposure and cell death in the cancer cells by activating CD47 signalling [14, 15], ii) block the ‘don’t-eat-me’ function of CD47, enabling phagocytosis [11], iii) enable greater access of antibodies to cancer cells [16], or iv) induction of antibody-dependent cell-mediated cytotoxicity (ADCC) [17]. However, the mechanism by which anti-CD47 antibodies affect cancers remains unclear and controversial [18], and this limits the development of these antibodies as therapeutic agents.

Acute lymphoblastic leukeamia (ALL) is characterized by the clonal expansion of a population of abnormal lymphocytes, known as lymphoblasts when they originate from immature cells as in precursor-B ALL, the most common childhood leukaemia. Cancers of mature B-cell phenotype (ALL and lymphoma, e.g. Burkitt’s) are also seen in childhood and more commonly in young adults. There is a need for new therapies for B-ALL and lymphomas that are more effective and/or cause less long-term morbidity in survivors than the current therapy using DNA damaging drugs [19].

Chao et al [13] showed that anti-CD47 antibodies induce phagocytosis of ALL lymphoblasts by macrophages in culture and clearance of lymphoblasts in mice, suggesting that the antibody might be used therapeutically for ALL. However, it remains unclear: a) how the antibodies are inducing phagocytosis, in particular whether the Fc domain is involved, and b) how the lymphoblasts die, in particular whether the lymphoblast are phagocytosed alive or dead, and thus whether phagocytosis is the cause of death. The answers to these questions are fundamental to the design and use of therapeutic antibodies. Weismann and colleagues’ concept of ‘programmed cell removal’ [20] of cancer cells by phagocytes as a result of antibody binding does not distinguish between clearance of live or dead cancer cells, and thus does not identify the cause of death.

In the work described here, we sought to determine: a) whether anti-CD47 antibodies could induce phagocytosis of pre-B-ALL lymphoblasts and mature B-cell blasts by macrophages, b) whether anti-CD47 antibodies induce phagocytosis of otherwise live cells and thereby result in their cell death by phagocytosis, and c) how anti-CD47 antibodies induce phagocytosis. We conclude that: anti-CD47 antibodies do induce phagocytosis of pre-B-ALL lymphoblast and mature B-cells and the induced cell death is by phagocytosis, i.e. phagoptosis. The Fc domain appears key to this phagoptosis and similar cell death is observed with antibodies directed against CD 10 and 19 suggesting that exploitation of this mechanism may have therapeutic potential.

## RESULTS

### Anti-CD47 antibody induces phagocytosis of B lymphoblasts by macrophages

**Figure 1:**
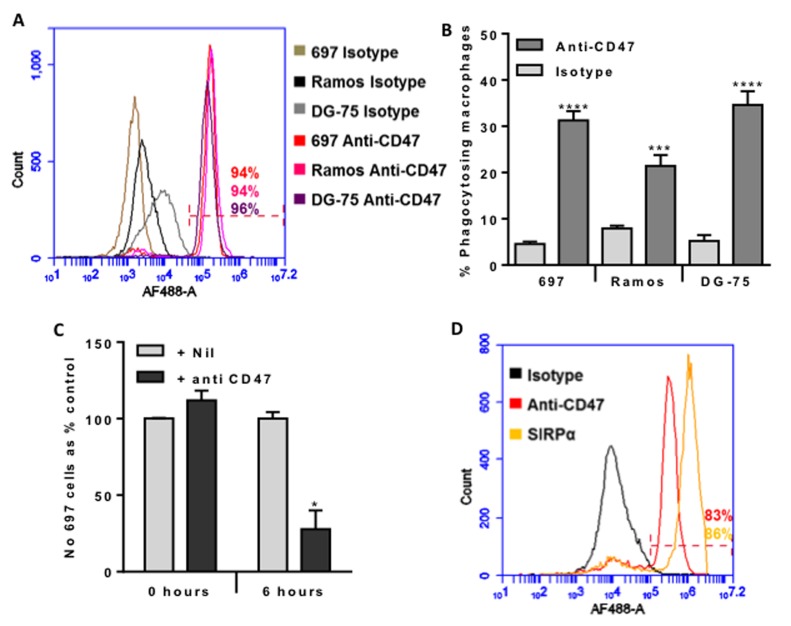
CD47 is expressed on the surface of malignant B-lymphocytes, and anti-CD47 antibodies increase their phagocytosis by macrophages dramatically reducing their numbers within 6 hours **A.** Antibody binding to 697 (pre-B lymphoblast cell line), Ramos and DG-75 (Burkitt lymphoma mature B lymphocyte cell lines) incubated with 10 µg/mL of isotype control or B6H12 anti-CD47 antibody, then AF488-conjugated secondary before analysing by flow cytometry. Representative example of 3 experiments. The % of each cell type binding the anti-CD47 antibody is given, and was gated on live cells (propidium iodide negative). **B.** U937 cells were matured into macrophages with 10nM PMA for 24 hours and further 48 hours incubation. Target B cells were stained with TAMRA, and macrophages with CFSE before co-incubating for 2 hours at 1:1 ratio with isotype or anti-CD47 B6H12 at 2 µg/mL. Phagocytosis assessed by measuring % of double positive macrophages. N≥4. Bars are mean ± SEM, *** / **** *p* < 0.001 / 0.0001, compared to isotype for that cell line. **C.** Phagocytosis assay prepared as above, but 697 stained with JC-1 and U937 with Höechst 33342. The number of free, live 697 cells was counted at 0 and 6 hours after addition of anti-CD47 antibody or nil. *N* = 4. **D.** Antibody binding to U937-derived macrophages incubated with 10 µg/mL of isotype control, B6H12 anti-CD47 antibody or anti-SIRPα antibody, then AF488-conjugated secondary before analysing by flow cytometry. Representative example of 3 experiments. The % of cells binding the anti-CD47 antibody and anti-SIRPα antibody are given, and was gated on live cells (propidium iodide negative). *N* = 3. Bars are mean ± SEM, * *p* < 0.05 compared with control.

CD47 has been reported to be overexpressed on a variety of cancer cell types in order to suppress phagocytosis of these cells. We tested whether CD47 was expressed on the surface of three different cell lines using a well characterised mouse antibody to human CD47 (clone B6H12). We found that CD47 was expressed on the surface of the three human B-cell lines used: 697 (pre-B-ALL), Ramos and DG-75 (both mature B-cells, Burkitt’s lymphoma)) (Figure 1A).

We next tested whether the antibody would induce phagocytosis of these cells by a human macrophage cell line (U937). The anti-CD47 antibody greatly increased phagocytosis by macrophages of all three cell lines as indicated by the uptake of fluorescently-labelled cells measured by flow cytometry (Figure 1B). The antibody also increased the phagocytosis by the macrophages of two other pre-B-ALL cell line NALM6 and REH, although to a lesser extent than the phagocytosis of 697 cells (Supplementary Figure 1).

We then selected the 697 lymphoblasts to co-incubate with macrophages at a 1:1 ratio, and determined whether the antibody-induced uptake would significantly deplete the population of lymphoblasts. 6 hours of co-culture resulted in no depletion of lymphoblast in the absence of antibody, but in the presence of antibody about 75% of the lymphoblasts were lost after 6 hours (Figure 1C). This suggests that the anti-CD47 antibody might be useful in the treatment of B-ALL.

CD47 on the surface of a cell is thought to block phagocytosis of that cell by engaging the SIRPα receptor on the surface of phagocytes, resulting in inhibition of phagocytosis. We therefore tested whether SIRPα and CD47 were expressed on the U937 macrophages, and found that both SIRPα and CD47 were both expressed on the macrophage surface (Figure 1D).

### The antibody does not directly kill lymphoblasts, but induces macrophage phagocytosis of live lymphoblasts, resulting in death by phagocytosis

**Figure 2:**
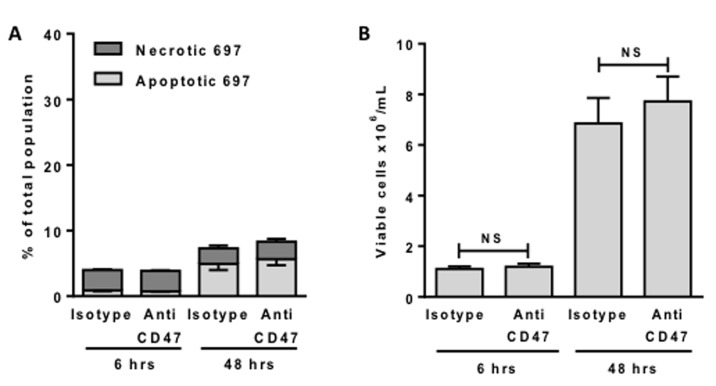
Anti-CD47 antibodies alone do not induce death or affect proliferation of 697 cells 697 cells were incubated for 6 or 48 hours with 2 μg/mL anti-CD47 or isotype control antibodies before staining and counting cells with annexin V for phosphatidylserine exposure (apoptosis) and propidium iodide (necrosis). Cells not staining with annexin V or propidium iodide were counted as viable. **A.** The % of necrotic and apoptotic cells is plotted. **B.** The number of viable cells in the same experiment is plotted. *N* = 3. Bars are mean ± SEM. NS = p > 0.05.

The anti-CD47 antibody might either a) kill the lymphoblasts, which could then induce their own phagocytosis via for example phosphatidylserine exposure, or b) induce the phagocytosis of otherwise viable lymphoblasts, resulting in their death by phagocytosis. We tested whether the anti-CD47 antibody induced apoptosis and/or necrosis of the lymphoblasts by measuring phosphatidylserine exposure with annexin V and necrosis with propidium iodide. However, there was no change in the proportion of apoptotic or necrotic lymphoblasts when exposed to anti-CD47 over 6 hours (Figure 2A). Thus the antibody alone did not induce apoptosis, necrosis or phosphatidylserine exposure of the lymphoblasts. Note also that >95% of lymphoblasts were viable (neither apoptotic or necrotic) after 6 hours of incubation in the presence or absence of antibody (Figure 2A). Even after 48 hours of incubation, the antibody induced no additional cell death or change in the number of viable cells (neither annexin V or propidium iodide positive), (Figures 2A & 2B), suggesting that the antibody has no effect on viability or proliferation in the absence of macrophages.

**Figure 3:**
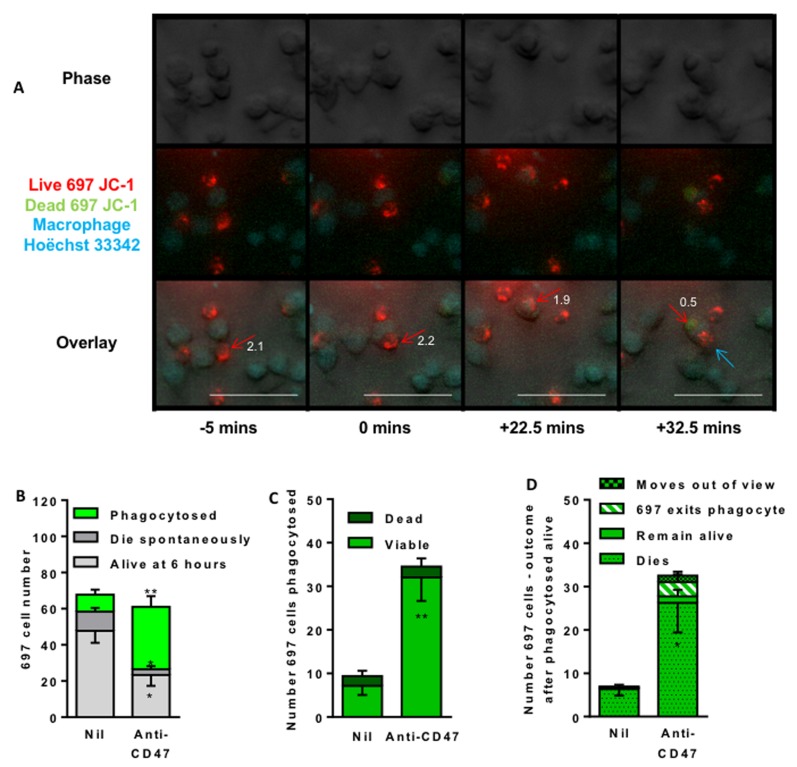
Time lapse imaging indicates that anti-CD47 antibody induces macrophage engulfment of live lymphoblasts 697 lymphoblasts were stained (mitochondria red/green) with JC-1 and U937 macrophages stained (nuclei blue) with Höechst 33342, and co-incubated at a 1:1 ratio and time-lapse imaged every 150 seconds for 6 hours with live/dead judged by change in red/green fluorescence of JC-1 and phagocytosis judged on phase images. **A.** Representative images to illustrate. The red arrow marks the first target cell, the blue arrow a second. Numbers by the red arrow is ratio of red:green fluorescence; a value < 1 suggests loss of cell viability. **B.** Outcome of 697 cells present in first frame. Four videos were analysed (Average 97 target cells and 96 macrophages in the first frame) following the fate of lymphoblasts present in first frame of video **C.** Number and viability (presence or absence of mitochondrial membrane potential) of 697 cells at time of phagocytosis (ie bright green bar of B). **D.** Outcome of those 697 cells apparently phagocytosed alive (i.e. bright green bar of C). “Dies” means loss of mitochondrial membrane potential, “live” means retains potential until end of video, “exits” means lymphoblast leaves macrophage, “moves out of view” means cell left the image frame. Data is shown as means ± SEM for at least 3 independent experiments performed in duplicate. * / ** *p* < 0.05 / 0.01 compared with control. Scale bar is 50 μM.)

The antibody might induce the macrophages to kill the lymphoblasts prior to phagocytosis. It is not known whether the cells induced to be phagocytosed by the anti-CD47 antibody are alive or dead at the time of engulfment, and therefore whether cell death precedes or follows engulfment. To test this, we stained the lymphoblasts with JC-1 to assess their mitochondrial membrane potential at the time of engulfment by the macrophages. In cells with normal, polarised mitochondria, the mitochondria fluoresce red, but this changes to green if the mitochondria depolarise. Almost all lymphoblast cells had intact mitochondrial membrane potential at the time of engulfment, but lost this potential about 30 mins after engulfment (Figure 3, Supplementary videos 1 and 2). When multiple videos were analysed, most of the lymphoblasts incubated with anti-CD47 antibody for 6 hours were phagocytosed by macrophages (Figure 3B). For each individual lymphoblast that was phagocytosed, over 90% had intact mitochondrial membrane potential at the time that they were being phagocytosed (Figure 3C), but after phagocytosis almost all lost this potential (Figure 3D), although a small but significant proportion re-emerged from the phagocytes alive. In summary, almost all the lymphoblasts were engulfed alive, but subsequently died.

**Figure 4:**
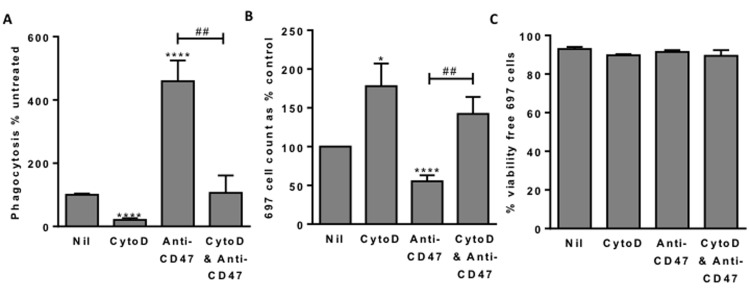
Blocking phagocytosis allows survival of 697 lymphoblast cells **A.** Phagocytosis assessed with flow cytometry after 2 hours as previously. 1 μM cytochalasin D (CytoD) incubated for 30 minutes with macrophages prior to addition of 697 cells. Anti-CD47 B6H12 used at 2 µg/mL. **B**. From the same samples, the number of free (unphagocytosed) 697 cells and **C**. their viability. *N* = 3, NS Not significant, * / **** *p* < 0.05 / 0.0001 compared with control, ## *p* < 0.01 as shown.

If the cause of cell death is phagocytosis, then inhibiting phagocytosis should prevent cell death. To test this we added cytochalsin D, which inhibits phagocytosis by blocking actin polymerisation. Addition of 1µM cytochalasin D inhibited the uptake of lymphoblasts into macrophages by about 80% (Figure 4A), and increased the number of free (unphagocytosed) lymphoblasts at the end of the co-incubation by about 80% (Figure 4B), and of those free lymphoblasts about 90% were viable (not apoptotic or necrotic) (Figure 4C). Thus blocking phagocytosis of the lymphoblasts left viable lymphoblasts, indicating that in the absence of inhibitor the cause of lymphoblast death was phagocytosis.

### Anti-CD47 antibody induces phagocytosis via the Fc domain

**Figure 5:**
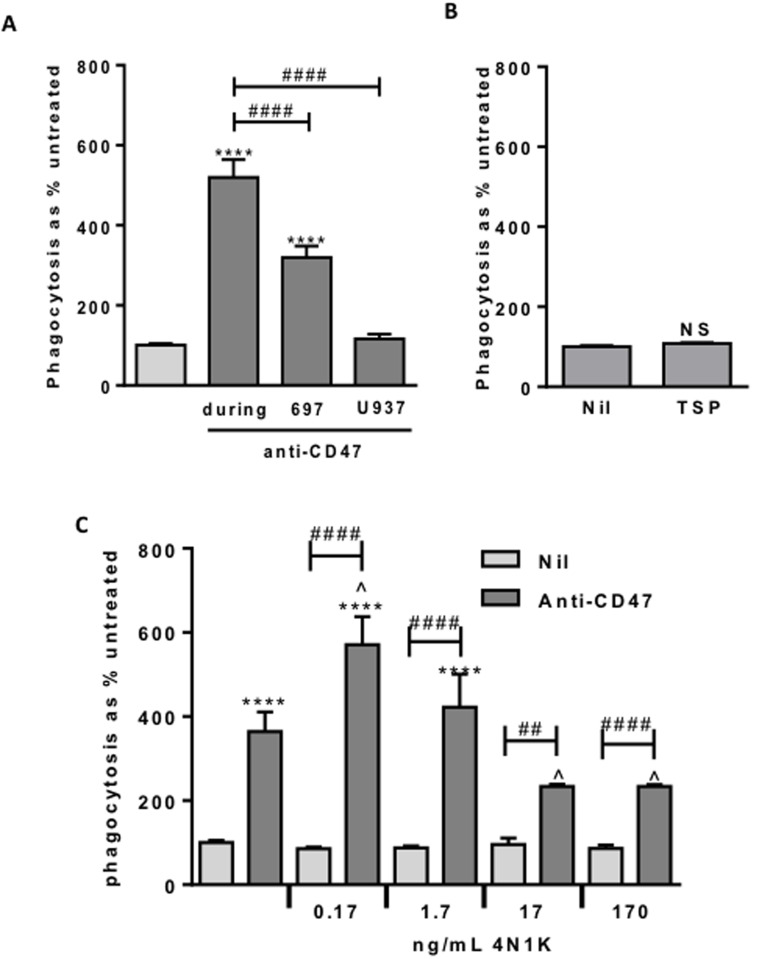
Anti-CD47 is only effective when bound to the target cells In the presence of anti-CD47, low dose 4N1K increases phagocytosis whereas higher dose inhibits. Phagocytosis assessed by flow cytometry after 2 hr co-incubation of 697 and U937-derived macrophages as previously. **A.** Anti-CD47 0.2 μg/mL, pre-incubated either with 697 cells or U937-derived macrophages for 30 minutes prior to washing off, or added at start of co-incubation (during). Similar results with 2 μg/mL, not shown. *N* = 3. **B.** Phagocytosis assay with the CD47-agonist thrombospondin (TSP) at 5 µg/mL. A range of doses showed the same result. *N* = 3. **C.** Phagocytosis assay with 4N1K ± anti-CD47 at 2 µg/mL. *N* = 3. ***** *p* < 0.0001 compared with control, ## / #### *p* < 0.01 / 0.0001 as indicated, ^ *p* < 0.05 compared with anti-CD47 alone. NS Not significant.

The anti-CD47 antibody bound to both the lymphoblasts and the macrophages (Figure 1). Thus the anti-CD47 antibody might be acting on the lymphoblasts or macrophages or both to induce phagocytosis. We tested whether incubating the anti-CD47 antibody with either cell type, followed by washing to remove unbound antibody could increase phagocytosis. Pre-incubation of lymphoblasts with antibody increased their subsequent phagocytosis three-fold (Figure 5A). Whereas pre-incubation of the macrophages with antibody had no effect on the subsequent phagocytosis of lymphoblasts (Figure 5A). For comparison, when the antibody was added to both cell types at the start of the co-incubation, then the phagocytosis was increased five-fold (Figure 5A). This indicates that antibody binds to the lymphoblasts and this then mediates uptake into the macrophages.

The anti-CD47 antibody might increase phagocytosis by activating CD47 receptor signalling. To test this, we added the CD47 receptor agonist thrombospondin-1 or a derivative peptide 4N1K, known to change the conformation of CD47 and strongly activate CD47 signalling [21]. Neither thrombospondin nor 4N1K affected macrophage phagocytosis of lymphoblasts in the absence of anti-CD47 antibody (Figure 5B & 5C), suggesting that activation of CD47 signalling is not sufficient to induce phagocytosis. Note however that we cannot be sure that thrombospondin activated CD47 in this experiment.

As 4N1K and the anti-CD47 antibody have the same target, CD47, we tested whether 4N1K affected the phagocytosis induced by the anti-CD47 antibody. We found that at low doses, 4N1K mildly increased the phagocytosis induced by anti-CD47 antibody, while at high concentrations 4N1K mildly inhibited the phagocytosis induced by anti-CD47 antibody (Figure 5C). As 4N1K had no effect on phagocytosis in the absence of the anti-CD47 antibody, this suggests that 4N1K binding to CD47 affects antibody binding or signalling via CD47.

**Figure 6:**
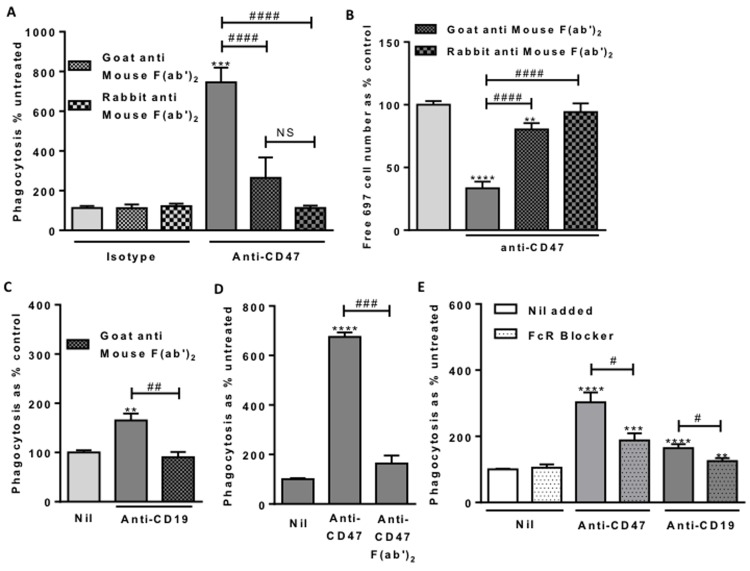
Macrophage Fc Receptor binding is necessary for anti-CD47 and anti-CD19 induced phagocytosis Phagocytosis assessed by flow cytometry after 2 hour co-incubation of 697 and macrophages as previously. Antibodies used at 2 μg/mL. **A.** Fc blocking F(ab’)_2_ fragments incubated with anti-CD47 for 1 hour prior to use. *N* = 3. **B.** From the same experiments, the numbers of free, unphagocytosed, 697 target cells. **C.** Fc blocking F(ab’)_2_ fragments incubated with anti-CD19 for an hour prior to use. *N* = 3. **D.** F(ab’)_2_ fragments of anti-CD47 B6H12 antibody at molar equivalent of 2 µg/mL compared with unfragmented antibody. N = 3. **E.** Fc Receptor blocker (FcR Blocker) pre-incubated with U937 cells for 30 minutes prior to assay. *N* = 3. ** / *** / **** *p* < 0.01 / 0.001 / 0.0001 compared with control, # / ### / #### *P* < 0.05 / 0.001 / 0.0001 as indicated.

The antibody might work through its antigen-binding Fab component, or its Fc receptor-binding Fc component, or it might require both. To investigate what role the Fc portion of the anti-CD47 antibody was playing, we blocked this with anti-Fc F(ab’)_2_ fragments that specifically bind and block mouse Fc. The uptake of lymphoblasts into macrophages induced by the anti-CD47 antibody was completely prevented by blocking its Fc using anti-mouse Fc F(ab’)_2_ fragments raised in goat or rabbit (Figure 6A). Similarly, the loss of free (unphagocytosed) lymphoblasts induced by antibody was prevented by the anti-Fc F(ab’)_2_ fragments (Figure 6B).

We next fragmented the anti-CD47 antibody and isolated the F(ab’)_2_ fragment lacking the Fc portion. This anti-CD47 F(ab’)_2_ fragment had no effect on phagocytosis, whereas the intact antibody stimulated phagocytosis of lymphoblasts by U937 macrophages (Figure 6D). This confirms that the Fc portion of the anti-CD47 antibody is required for the phagocytosis.

In order to test whether the anti-CD47 antibody would also induce phagocytosis of lymphoblasts by primary human macrophages, we isolated monocytes from fresh human blood, and differentiated these into macrophages, which were then incubated with 697 lymphoblasts ± the anti-CD47 antibody. The antibody increased the phagocytosis of lymphoblasts by primary macrophages, although not to the same extent as with U937 macrophages (Supplementary Figure 1C). As with U937 macrophages, the uptake of lymphoblasts into primary macrophages induced by the anti-CD47 antibody was prevented by blocking its Fc domain using anti-mouse Fc F(ab’)_2_ fragments raised in goat and F(ab’)_2_ fragments derived from anti-CD47 did not induce phagocytosis (Supplementary Figure 1C).

The Fc domain of an antibody can induce phagocytosis via phagocytic Fc receptors on macrophages. We therefore blocked these receptors with a human Fc receptor binding inhibitor, and found that this inhibited anti-CD47-induced phagocytosis (Figure 6E). This demonstrates that Fc receptors are necessary for anti-CD47-driven phagocytosis.

### Anti-CD10 and CD19 antibodies can also induce phagocytosis of lymphoblasts

**Figure 7:**
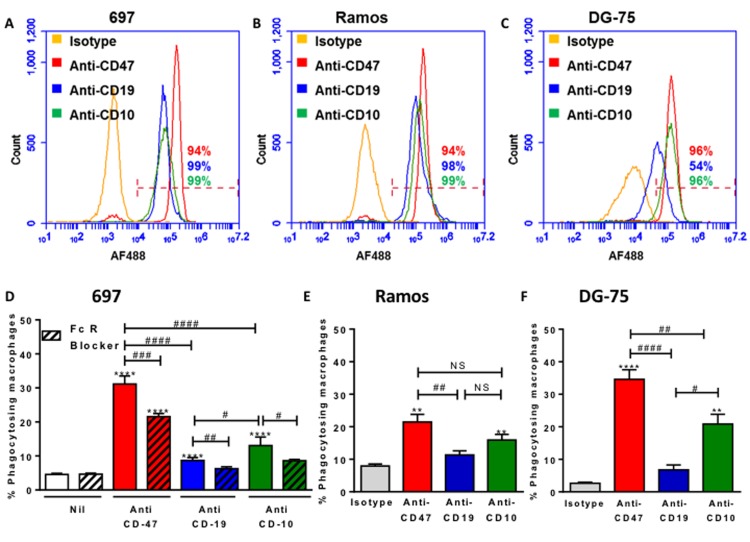
Antibodies to three cell surface expression markers increases phagocytosis and the potency of antibodies to induce phagocytosis is related to expression levels **A.**-**C.** Cells were labelled with 10 μg/mL of antibody, then AF488-conjugated secondary before analysing live cells by flow cytometry. Representative plots of three repeats. **D.**-**F.** Target cells were stained with TAMRA, and macrophages with CFSE then coincubated for 2 hours at 1:1 ratio with isotype or antibody. FcR Blocker is Fc receptor blocker, pre-incubated with U937 cells for 30 minutes prior to assay. Anti-CD47 and anti-CD19 at 2 µg/mL, anti-CD10 at 5 µg/mL. *N* = 3. * / ** / *** / **** *p* < 0.05 / 0.01 / 0.001 / 0.0001, compared with control, # as indicated, NS not significant.

If the anti-CD47 antibody induces phagocytosis of the lymphoblast by binding both CD47 on lymphoblasts and Fc receptors on macrophages, then any other antibody that binds lymphoblasts should induce phagocytosis by the same means. We compared the expression level of CD47, CD10 and CD19 on 697, Ramos and DG-75 cells, and the extent to which these antibodies induced phagocytosis (Figure 7). The degree of phagocytosis roughly reflected the surface expression levels of the antigens, although the anti-CD19 antibody was relatively ineffective at inducing phagocytosis of Ramos cells (Figure 7E). This suggests that rather than needing to target a specific pathway, such as the CD47-SIRPα pathway, phagocytosis of cancer cells can be increased simply by targeting cell surface markers that are highly expressed. And such antibodies work by binding antigen on target cells and activating phagocytic Fc receptors on phagocytes. This is supported by our finding that phagocytosis was prevented by blocking either the Fc domain of the antibodies (Figure 6A & 6C) or the Fc receptors (Figures 6C, 6E & 7D).

## DISCUSSION

Antibodies are rapidly becoming one of the most effective therapeutic options for cancer, but the mechanism of action is often unclear [22]. The mechanism matters to the design of antibodies and how they are used [23, 24]. The anti-CD47 antibody has been suggested to induce phagocytosis by several potential mechanisms: 1) induction of phosphatidylserine exposure and/or apoptosis in the cancer cells by activating CD47 signalling, 2) blocking the ‘don’t-eat-me’ function of CD47, enabling phagocytosis, 3) induction of antibody-dependent cell-mediated cytotoxicity (ADCC) and/or 4) induction of cell death by phagocytosis. We have shown above in our system that option 1 does not apply as: a) activating CD47 with thrombospondin-1 or 4N1K had no effect on phagocytosis in the absence of the antibody, and b) the antibody did not induce phosphatidylserine exposure, apoptosis or cell death in the absence of macrophages. Option 2 does not apply as we found that antibody-induced phagocytosis was prevented by either: a) blocking the Fc domain of the antibody, b) removing the Fc domain of the antibody, or c) blocking Fc receptors. Thus blocking CD47 is not sufficient to induce phagocytosis in this system. Our data are consistent with option 4, not 3, as a) the cells were alive when phagocytosed, and died after phagocytosis, and b) blocking phagocytosis prevented cell death.

In general, antibody binding can induce death of a cell by: i) changing the activity of the specific antigen, ii) induction of antibody-dependent cell-mediated cytotoxicity (ADCC), e.g. by complement-mediated lysis, NK cells or cytotoxic T cells and/or iii) induction of antibody-dependent cellular phagocytosis (ADCP) [22]. Although ADCP can be an important mechanism of action of therapeutic antibodies, it is again unclear whether phagocytosis is the cause or consequence of cell death. And this issue also determines whether adaptive immunity to cancer via antibodies [25, 26] is in part mediated by phagoptosis i.e. cell death as a result of phagocytosis.

The role of the Fc portion of anti-CD47 has been investigated to a limited extent previously. F(ab’)_2_ fragments (lacking the Fc domain) of the B6H12 anti-CD47 clone were found sufficient to induce phagocytosis of Burkitt’s lymphoma cell line (Raji) by mouse macrophages [17], but not the phagocytosis of breast cancer cells by human neutrophil phagocytosis [16]. Several studies showed that the function blocking B6H12 clone increased phagocytosis, whereas the non-function blocking 2D3 anti-CD47 clone did not induce phagocytosis [13, 17, 25, 27], suggesting that CD47 function blocking is required, not just binding. However, it is unclear that the 2D3 clone binds with the same affinity as the B6H12 clone to either CD47 or Fc receptors.

Our data are consistent with the anti-CD47 antibody killing cancer cells by antibody-dependent cellular phagocytosis (ADCP) [22]. And our results suggest that cancer cells may be targeted for phagocytosis simply by adding human antibodies that bind cancer cell surface markers that are highly expressed. CD47 and CD10 appear to be suitable targets for pre-B-ALL and mature B-cell cancer. Anti-CD10 and anti-CD19 antibodies have already been used to deplete B-ALL lymphoblasts *in vivo* [28, 29], and ADCP might contribute to this antibody’s mechanism of action. If cancer cells express novel antigens on their surface then endogenous antibodies will bind to them, and the current work suggest such antibodies might contribute to immune defence by inducing phagoptosis.

4N1K is a peptide consisting of the 8 amino acid residues (RFYVVMWK) at the N terminal of thrombospondin-1, which is thought to bind two different sites on CD47 [30]. CD47 binds SIRPα from one of these sites, and this can be blocked by the B6H12 anti-CD47 clone [31]. 4N1K is known to induce a conformational change in CD47 that can increase phagocytosis [21], and this might explain why low levels of 4N1K can increase anti-CD47-induced phagocytosis, as a result of increasing the binding of the antibody to CD47. Higher levels 4N1K might block anti-CD47-induced phagocytosis by blocking antibody binding at the second binding site. Note however that very high concentrations of 4NIK can change antibody binding to cells independent of CD47 [32]. A potential application of the finding that low levels of 4N1K can enhance anti-CD47-induced phagocytosis is that this might be used to enhance anti-CD47 cancer therapy *in vivo*. However, in terms of the mechanism of action of the antibodies, the important point is that activating CD47 is not sufficient to induce phagocytosis.

In conclusion, this work demonstrates: that anti-CD47 causes macrophage phagocytosis of live lymphoblasts and their subsequent death, that other antibodies can also induce phagocytosis of B lymphoblasts and mature B malignant cells, and that antibody induced phagocytosis is dependent on an intact Fc domain. This work suggests that phagoptosis may contribute to both immune defence against cancer and potential therapies.

## MATERIALS AND METHODS

### Cell culture

697 were established in 1979 from a 12 year old boy with relapsed acute leukaemia (ALL). They are a pre-B lymphoblastic cell line and carry the genetic translocation t(1;19)(q23;p13) [33]. 697 cells stably infected with control retrovirus (697-Neo), or recombinant Bcl-2 containing retrovirus (697- BCL2) were kindly provided by Professor Miyashita [34]. U937 cells were a gift from Professor Goodall, University of Cambridge, School of Clinical Medicine. 697 and U937 were cultured in RPMI 1640 (Life Technologies) supplemented with 10% foetal bovine serum (FBS, Life Technologies, UK) and 1% penicillin/streptomycin (Sigma) at 37°C, 5% CO_2_. To induce differentiation, U937 were cultured on coated wells (poly-L-lysine 0.001% in PBS) for 3 days, the first 24 hours with phorbol myristate acetate (PMA, 10 nM, Sigma).

### Reagents

Anti-CD47 monoclonal antibody (mAb) B6H12, anti-CD19 mAb HIB19, anti-CD10, mouse IgG1 kappa isotype control and human Fc receptor binding inhibitor were from eBioscience. The latter inhibitor blocks binding of antibodies to Fc receptors on U937 macrophages (www.ebioscience.com/human-fcr-binding-inhibitor-purified.htm). F(ab’)_2_ fragments (Stratech) were used in 5:1 molar ratio fragment:antibody, incubated 30 minutes prior to use with antibodies. Cell dyes were from Sigma, except JC-1 from Life Technologies. 4N1K was from AnaSpec, thrombospondin from Athens Research and Technology. Cytochalasin D (Sigma) applied to stained U937 cells 30 minutes prior to phagocytosis assay.

### Phagocytosis assays

Target (lymphoblasts) and effector (matured U937 cells) were stained then co-cultured in a 1:1 ratio. Phagocytosis was either assessed with fluorescence microscopy (Leica DMI6000 CS microscope (HCX P1 Fluotar 20x/0.40 dry objective (1hr) or 40x (Time lapse)), or flow cytometry (Accuri C6, BD Biosciences). For microscope images, engulfment was judged by looking for fragments of target cell stain contained within an effector cell, or judged on overlay images for time lapse. Analysis was performed using Image J, with four fields of view taken per well, performed in duplicate. Phagocytosis index is number of phagocytosing macrophages / Total number macrophages per 100 macrophages on field. For flow cytometry, cells were gated tightly on width to exclude doublets. Gating on CFSE-stained U937 cells, the shift into TAMRA FL1 gate was assessed. Single stained controls were used to set gates.

### Cell surface expression

B cells were blocked with 1% BSA then incubated with Fc-blocked primary antibodies, washed and incubated with Alexa Fluor-conjugated secondary antibodies and analysed with Accuri C6. To identify live cells, they were co-stained with either PI or Annexin-V. Control staining was done using isotype-matched IgG and secondary antibodies alone.

### Primary human macrophages

Human peripheral blood derived monocytes were isolated from buffy coat (white blood cell fraction), obtained from the UK National Blood Service, by centrifugation on a Ficoll gradient, and subsequent selection of those cells adhering to plastic. Macrophages were matured from these monocytes over 8-9 days using 50ng/mL M-CSF. Phagocytosis was assessed by flow cytometry after 2 hour co-incubation with 697 cells as previously. Antibodies were used at 5 μg/mL, or molar equivalent for fragmented antibody. Goat anti-mouse or rabbit anti-mouse F(ab’)_2_ fragments were pre-incubated with antibody for 1 hour prior to use.

### Statistics

All analyses were performed with GraphPad Prism 6, with one-way Anova with post-hoc Sidak analysis unless stated otherwise.

## SUPPLEMENTARY MATERIALS FIGURE



## SUPPLEMENTARY VIDEOS




