# Supplementary material for: Anti-CD47 antibodies induce phagocytosis of live, malignant B cells by macrophages via the Fc domain, resulting in cell death by phagoptosis
Source: Oncotarget. 2017 Jun 15;8(37):60892–903. doi: 10.18632/oncotarget.18492 (PMC5617392; doi:10.18632/oncotarget.18492)
Supplement: Supplementary file 1 [file oncotarget-08-60892-s001.pdf]

Anti-CD47 antibodies induce phagocytosis of live, malignant B cells by macrophages *via* the Fc domain, resulting in cell death by phagoptosis

Supplementary Material

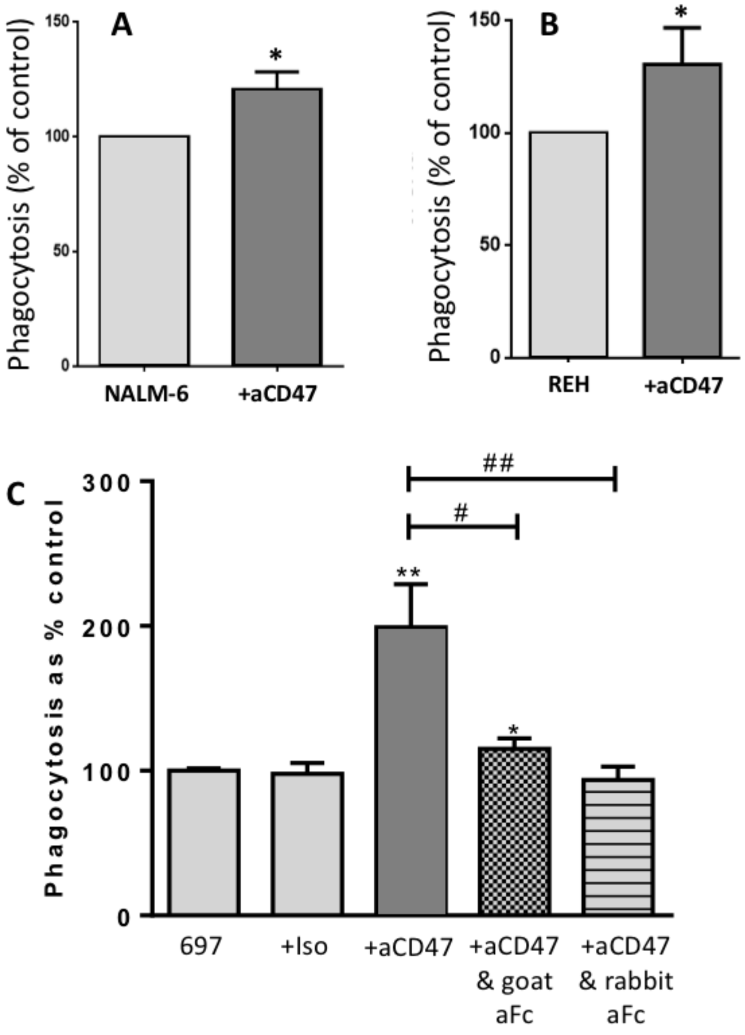

Supplementary Figure 1. Anti-CD47 antibody induces phagocytosis of pre-B-ALL cell lines NALM-6 and REH by U937 macrophages, and anti-CD47 induced phagocytosis of 697 cells by primary human macrophages requires Fc domain. A) NALM-6 were incubated with U937 ± 50 µg/ml of anti-CD47 (aCD47). n=3. * p<0.05 Mann-Whitney U. B) REH were incubated with U937 ± 10 µg/ml of anti-CD47 (aCD47). n=4. * p<0.05. C) Primary macrophages matured from peripheral blood derived monocytes with 697 cells for 2 hours ± 5 µg/mL anti-CD47 antibody, ± molar equivalent of goat anti-mouse or rabbit anti-mouse anti-Fc F(ab')2 fragments (aFc) pre-incubated with antibody for 1 hour prior to use. N=4, performed in duplicate or triplicate. * / ** p < 0.05 / 0.01 compared with untreated, # / ## p < 0.05 / 0.01 as indicated.

For Supplementary Videos see in Supplementary Files.
